# Biogas production using anaerobic groundwater containing a subterranean microbial community associated with the accretionary prism

**DOI:** 10.1111/1751-7915.12179

**Published:** 2014-09-29

**Authors:** Kyohei Baito, Satomi Imai, Makoto Matsushita, Miku Otani, Yu Sato, Hiroyuki Kimura

**Affiliations:** 1 Department of Geosciences, Graduate School of Science, Shizuoka UniversityShizuoka, Japan; 2 Center for Integrated Research and Education of Natural Hazards, Shizuoka UniversityShizuoka, Japan; 3 PRESTO, Japan Science and Technology Agency (JST)Kawaguchi, Saitama, Japan

## Abstract

In a deep aquifer associated with an accretionary prism, significant methane (CH_4_) is produced by a subterranean microbial community. Here, we developed bioreactors for producing CH_4_ and hydrogen (H_2_) using anaerobic groundwater collected from the deep aquifer. To generate CH_4_, the anaerobic groundwater amended with organic substrates was incubated in the bioreactor. At first, H_2_ was detected and accumulated in the gas phase of the bioreactor. After the H_2_ decreased, rapid CH_4_ production was observed. Phylogenetic analysis targeting 16S rRNA genes revealed that the H_2_-producing fermentative bacterium and hydrogenotrophic methanogen were predominant in the reactor. The results suggested that syntrophic biodegradation of organic substrates by the H_2_-producing fermentative bacterium and the hydrogenotrophic methanogen contributed to the CH_4_ production. For H_2_ production, the anaerobic groundwater, amended with organic substrates and an inhibitor of methanogens (2-bromoethanesulfonate), was incubated in a bioreactor. After incubation for 24 h, H_2_ was detected from the gas phase of the bioreactor and accumulated. Bacterial 16S rRNA gene analysis suggested the dominance of the H_2_-producing fermentative bacterium in the reactor. Our study demonstrated a simple and rapid CH_4_ and H_2_ production utilizing anaerobic groundwater containing an active subterranean microbial community.

## Introduction

Accretionary prisms are distributed over the convergent plate boundary, where oceanic and continental crusts collide, and found in large regions of the world, e.g. Japan, Taiwan, Indonesia, Peru, Chile and New Zealand, and Alaska and Washington in the United States (Kano *et al*., 1991; Davis *et al*., 1998; Fagereng, 2011; Hervé *et al*., 2013). These geologic features, also known as accretionary wedges, consist of a thick sediment accreted onto the non-subducting continental crust. The sediment is derived from marine sediment on the subducting oceanic crust and contains abundant organic matter (Tanabe and Kano, 1996). The sediment has layers of water-bearing permeable sandstone and no water-bearing impermeable mudstone. Groundwater is mainly recharged by rainfall which infiltrates into outcrops or faults, then flows down through the permeable sandstone and is anaerobically reserved in a deep aquifer. Furthermore, it has been reported that a large amount of the natural gas [methane (CH_4_), > 97%] is present in deep aquifer associated with the accretionary prism (Igari and Sakata, 1989; Sakata *et al*., 2012).

Kimura and colleagues (2010) performed a series of geochemical and microbiological studies of anaerobic groundwater and natural gas obtained from a deep aquifer associated with an accretionary prism in southwest Japan. Stable carbon isotopic analysis of CH_4_ in the natural gas and total dissolved inorganic carbon in the groundwater, mainly bicarbonate, suggested that CH_4_ in the deep aquifer is generated by a biogenic process. Archaeal 16S ribosomal ribonucleic acid (rRNA) gene analysis revealed the dominance of hydrogenotrophic methanogens in the anaerobic groundwater. A high potential of CH_4_ production by hydrogenotrophic methanogens was shown in enrichment cultivation using anaerobic groundwater amended with hydrogen (H_2_) and carbon dioxide (CO_2_). Furthermore, bacterial 16S rRNA gene analysis showed that H_2_-producing fermentative bacteria were included in anaerobic groundwater obtained from the deep aquifer. Anaerobic incubation using groundwater amended with organic substrates suggested a high potential of H_2_-producing fermentative bacteria.

To date, there is a great amount of literature on bioreactors for biogas production by using such resources as industrial and municipal wastewaters, animal manures, agricultural crop straws and food and green wastes (e.g. Ishii *et al*., 2005; Hori *et al*., 2006; Li *et al*., 2013). However, there has been no report of a bioreactor using anaerobic groundwater associated with an accretionary prism. In this study, therefore, we developed bioreactors for producing both CH_4_ and H_2_ using anaerobic groundwater containing an active subterranean microbial community. The bioreactors performance was evaluated through volumetric biogas production rates and times repeated biogas production. The findings may be used for the development of a novel bioenergy production system that combines the bioreactors for CH_4_ and H_2_ production and a ‘natural subterranean methane reactor’ in a deep aquifer associated with an accretionary prism.

## Results and discussion

### Physical and chemical data of groundwater and natural gas

We collected groundwater and natural gas samples from a deep well situated in Shimada, Shizuoka Prefecture, Japan (Figs S1 and S2). Physical parameters and chemical compositions of the groundwater and natural gas were determined (Table S1). The groundwater left the deep well at a temperature of 41.0°C, pH of 8.2 and oxidation-reduction potential (ORP) of − 270 mV. The microbial cell density in the groundwater was 3.3 × 10^4^ cells ml^−1^. In the natural gas, CH_4_ was the predominant component. The physicochemical parameters and microbial cell density were in good agreement with those reported in the previous studies (Kimura *et al*., 2010; Kaneko *et al*., 2014).

### Performance of the bioreactor for CH_4_ production

**Figure 1:**
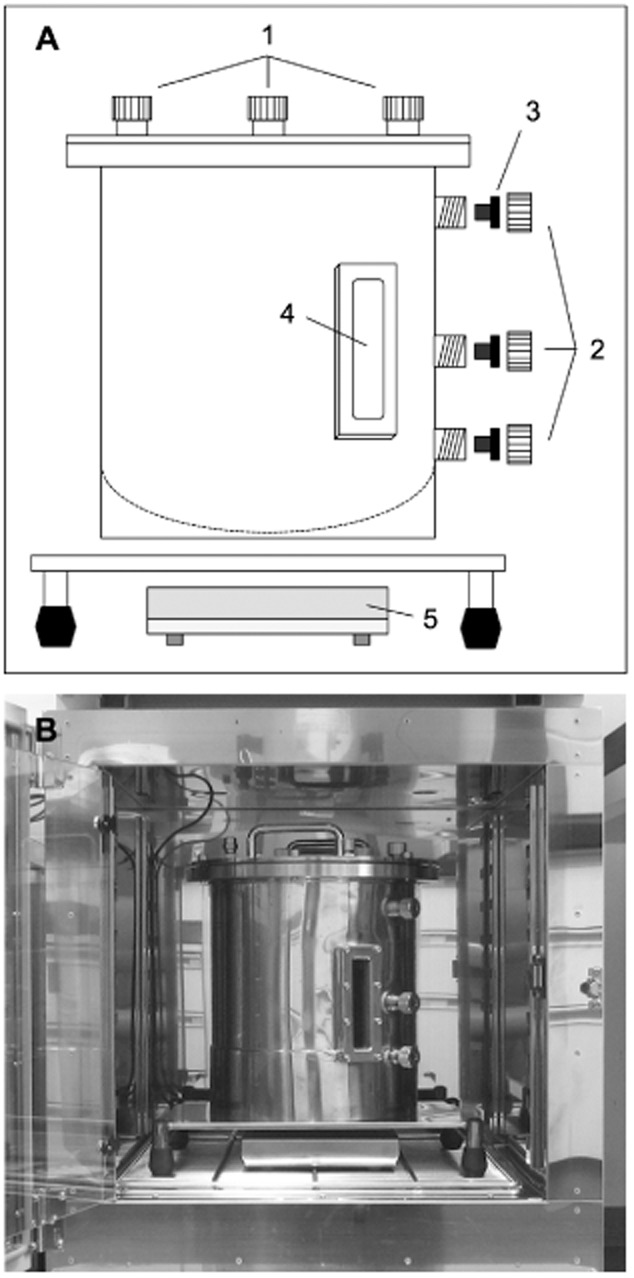
Structure of the bioreactor (A) and photo of the bioreactor placed in an incubator (B). 1, influent ports; 2, sampling ports; 3, butyl rubber stoppers; 4, viewing window; 5, magnetic stirrer.

**Figure 2:**
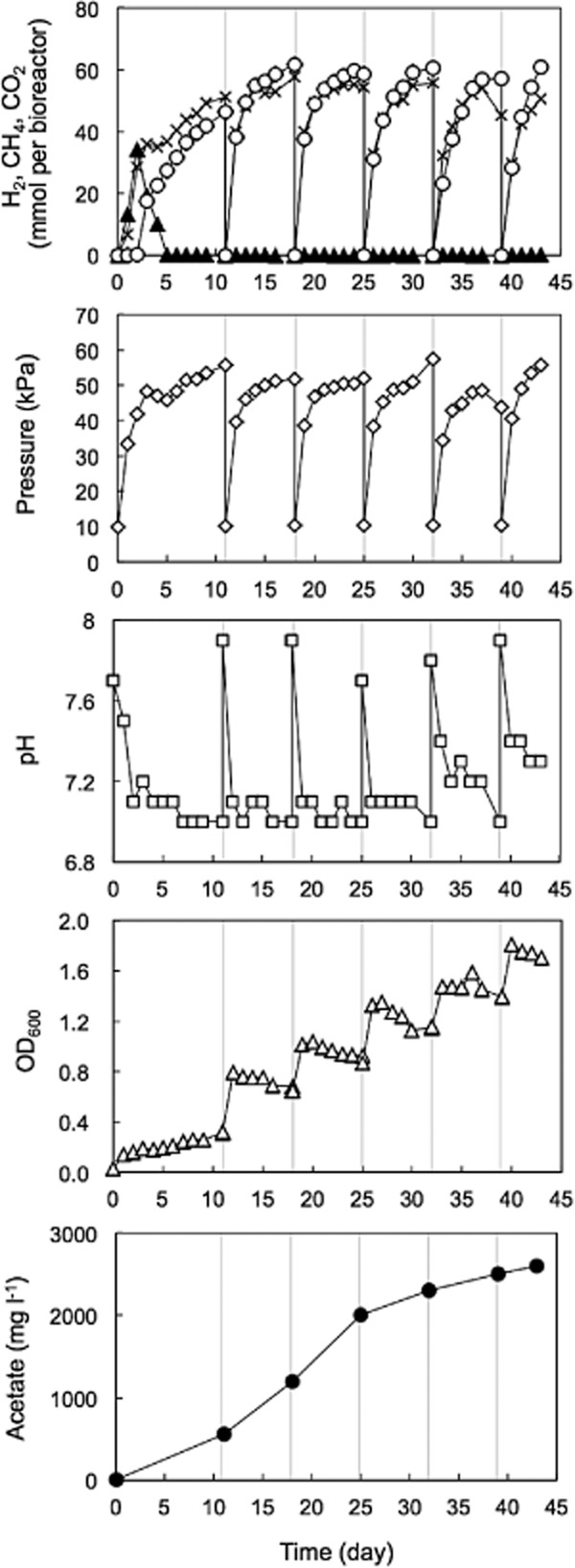
Dynamics of the biogas and reactor in the bioreactor for CH_4_ production using anaerobic groundwater amended with 0.22% YPG: ▲, H_2_; ○, CH_4_; ×, CO_2_; ◇, pressure; □, pH; △, OD_600_; ●, acetate. Gray lines indicate YPG supplement and biogas removal by bubbling using pure N_2_.

The groundwater sample was anaerobically collected from the deep well and directly poured into the bioreactor (Fig. 1A) through the influent port using a sterile silicone tube (Fig. S3). In order to produce CH_4_, the anaerobic groundwater amended with yeast extract, peptone and glucose (YPG) medium was incubated at 55°C (Fig. 1B). Initially, H_2_ was detected within 24 h and accumulated in the gas phase of the bioreactor. Hydrogen then decreased to below the detection limit. Following the disappearance of the H_2_, CH_4_ production began to be observed. Methane increased to 46 mmol in the bioreactor amended with 0.22% YPG (Fig. 2) and 174 mmol in the bioreactor amended with 1.0% YPG (Fig. S4). The pressure of the gas phase was increased to 55.7 kPa and 129 kPa in the bioreactor supplemented with 0.22% YPG and 1.0% YPG respectively.

When the biogas production rate became low, YPG medium was added to the bioreactor. After biogas generated in the bioreactor was removed using pure nitrogen gas (N_2_), the bioreactor was incubated at 55°C again without groundwater exchange and pH adjustment. In the second incubation, rapid CH_4_ production was observed, whereas H_2_ was below the detection limit. Methane production was performed at least six times in the bioreactor operation using 0.22% YPG (Fig. 2) and at least four times in the bioreactor operation using 1.0% YPG (Fig. S4). The pH was maintained approximately 7.1 and 6.6 in the bioreactor operations using 0.22% and 1.0% YPG respectively. The optical density at 600 nm (OD_600_) for monitoring the microbial biomass and the acetate concentration increased with the incubation time.

The maximum CH_4_ production rate was 17.8 mmol liter^−1^ day^−1^ in the bioreactor amended with 1.0% YPG (Table 1). This value was comparable to the CH_4_ production rates that have been reported in previous studies (Liu *et al*., 2013; Carrillo-Reyes *et al*., 2014; Zhong *et al*., 2014).

**Table 1:** Biogas production rates in bioreactors for CH_4_ and H_2_ production

	YPG conc. (%)	Maximum pressure (kPa)	Biogas production rate (mmol liter^−1^ day^−1^)
CH_4_ production	0.22	55.7	7.48
	1.0	129	17.8
H_2_ production	0.22	46.5	6.08
	1.0	58.6	0.86

### Microbial community in the bioreactor for CH_4_ production

A total of 49 clones in the archaeal 16S rRNA gene clone library were sequenced and found to belong to only one operational taxonomic unit (OTU; MET-BR-A01) (Table 2). MET-BR-A01 was closely related to the 16S rRNA gene from the thermophilic hydrogenotrophic methanogen *Methanothermobacter thermautotrophicus* (99% similarity), which uses H_2_ and CO_2_ for methanogenesis (Zeikus and Wolfe, 1972).

**Table 2:** Archaeal and bacterial 16S rRNA gene sequences derived from microbial communities in bioreactors for CH_4_ and H_2_ production

OTU	No. of clones	Phylogenetic class	Closest cultivated species (% similarity)
Bioreactor for CH_4_ production			
*Archaea*			
MET-BR-A01	49	*Methanobacteria*	*Methanothermobacter thermautotrophicus (99)*
Total	49		
*Bacteria*			
MET-BR-B01	79	*Clostridia*	*Tepidanaerobacter syntrophicus (93)*
MET-BR-B02	5	*Clostridia*	*Acetivibrio cellulolyticus (91)*
MET-BR-B03	4	*Clostridia*	*Gelria glutamica (98)*
MET-BR-B04	1	*Clostridia*	*Tepidanaerobacter syntrophicus (92)*
MET-BR-B05	1	*Clostridia*	*Desulfosporosinus acidiphilus (95)*
Total	90		
Bioreactor for H_2_ production			
*Bacteria*			
HYD-BR-B01	47	*Clostridia*	*Thermotalea metallivorans (99)*
HYD-BR-B02	10	*Clostridia*	*Tepidanaerobacter syntrophicus (83)*
HYD-BR-B03	9	*Flavobacteria*	*Schleiferia thermophila (88)*
HYD-BR-B04	5	*Clostridia*	*Desulfotomaculum putei (99)*
HYD-BR-B05	5	*Clostridia*	*Desulfotomaculum putei (89)*
HYD-BR-B06	4	*Clostridia*	*Thermosyntropha lipolytica (94)*
HYD-BR-B07	4	*Clostridia*	*Thermotalea metallivorans (99)*
HYD-BR-B08	3	*Clostridia*	*Oxobacter pfennigii (92)*
HYD-BR-B09	3	*Clostridia*	*Clostridium thermosuccinogenes (93)*
HYD-BR-B10	1	*Clostridia*	*Anaerobranca horikoshii (98)*
Total	91		

In a bacterial 16S rRNA gene clone library, a total of 90 clones were sequenced and divided into five OTUs (MET-BR-B01 to MET-BR-B05) (Table 2). The coverage of the clone library was 98%. The most abundant OTU, MET-BR-B01, was closely related to the 16S rRNA gene from the thermophilic fermentative bacterium *Tepidanaerobacter syntrophicus* (93% similarity), which is known to degrade organic matter to H_2_ and CO_2_ and produce CH_4_ in a coculture with the hydrogenotrophic methanogen *M. thermautotrophicus* (Sekiguchi *et al*., 2006). The MET-BR-B01 clones accounted for 88% of the clones in the clone library. MET-BR-B02 showed the highest similarity to the 16S rRNA gene from the cultured bacterium *Acetivibrio cellulolyticus* (91% similarity), which degrades cellulose to H_2_ and CO_2_ and produces CH_4_ in a coculture with a methanogenic archaea (Khan, 1980), and accounted for 5.6% of all the clones in the library. MET-BR-B03 was closely related to the 16S rRNA gene from the glutamate-degrading bacterium *Gelria glutamica* (98% similarity), which is also known to produce CH_4_ in coculture with hydrogenotrophic methanogens (Plugge *et al*., 2002). The MET-BR-B03 clones accounted for 4.4% of the clones in the library. The remaining low-abundance OTUs showed the closest matches to the 16S rRNA genes from anaerobic bacteria of the class *Clostridia* (MET-BR-B04 and MET-BR-B05).

**Figure 3:**
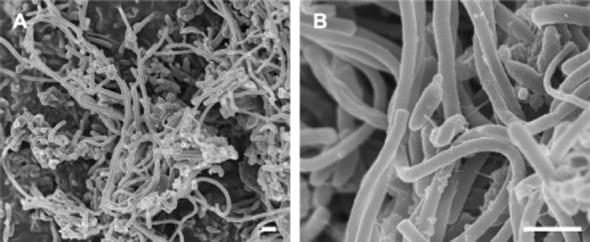
Scanning electron micrographs of microbial cells derived from the reactor for CH_4_ production. Bar, 1 μm.

Scanning electron microscopy showed the dominance of long, irregularly curved cells in the culture fluid for CH_4_ production (Fig. 3A). In addition to the long cells, relatively thick, rod-shaped cells were observed in the reactor. We also found flagellum-like filaments connecting the long cells and rod-shaped cells, particularly in large cell aggregates (Fig. 3B). Although the phylogenetic position of the long cells was not determined, they were considered likely to be the hydrogenotrophic methanogen *M. thermautotrophicus* based on their morphological characteristics (Zeikus and Wolfe, 1972). A previous study suggested that the flagellum-like filaments provided ‘interspecies electron/hydrogen transfer’ in a syntrophic consortium established by fermentative bacteria and methanogenic archaea (Ishii *et al*., 2005; Shimoyama *et al*., 2009; Hillesland and Stahl, 2010; Walker *et al*., 2012). The results underscore that the microbial syntrophy of *T. syntrophicus* and *M. thermautotrophicus* derived from the anaerobic groundwater contributes to the CH_4_ production in the bioreactor.

### Performance of the bioreactor for H_2_ production

**Figure 4:**
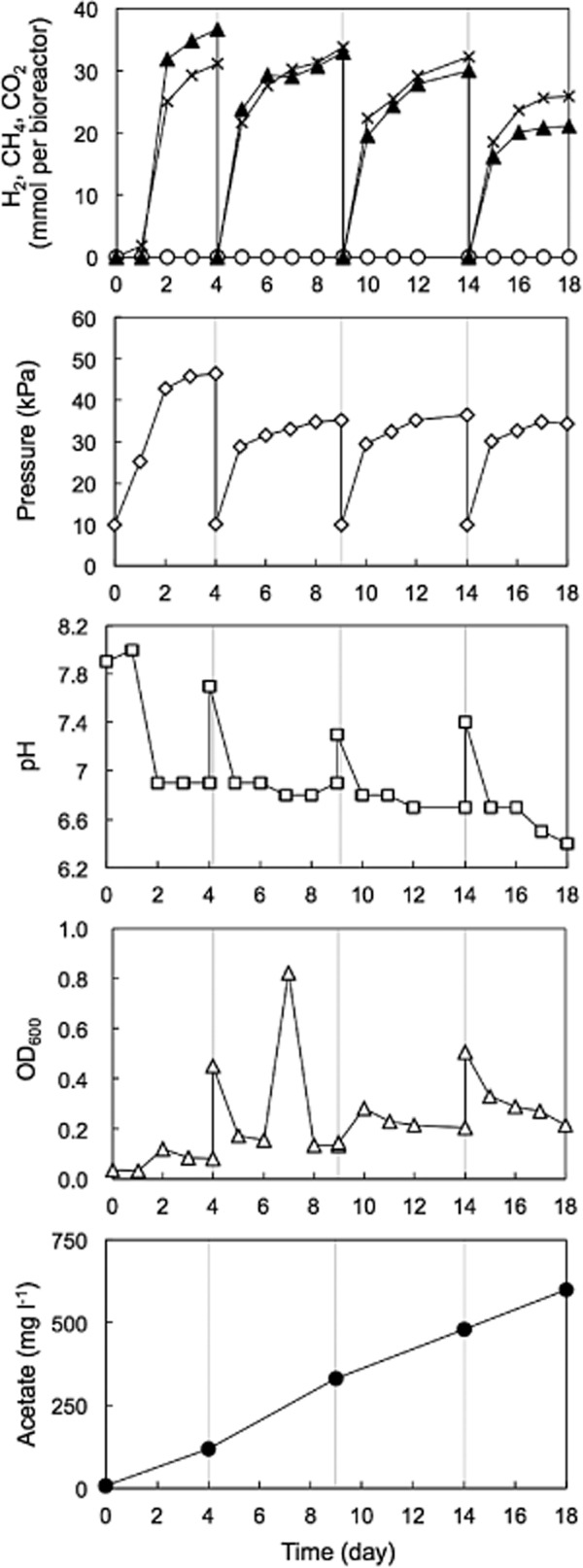
Dynamics of the biogas and reactor in the bioreactor for H_2_ production using anaerobic groundwater amended with 0.22% YPG-plus-20 mM BES: ▲, H_2_; ○, CH_4_; ×, CO_2_; ◇, pressure; □, pH; △, OD_600_; ●, acetate. Gray lines indicate YPG supplement and biogas removal by bubbling using pure N_2_.

For H_2_ production, anaerobic groundwater amended with YPG medium and an inhibitor of methanogens [2-bromoethanesulfonate, (BES)] was incubated at 55°C. Hydrogen was detected from the gas phase of the bioreactor within 48 h. Then, H_2_ accumulated and rose to approximately 35 mmol in both bioreactors amended with 0.22% and 1.0% YPG. The pressure of the gas phase increased to 47 kPa in the bioreactor amended with 0.22% YPG (Fig. 4) and 59 kPa in the bioreactor amended with 1.0% YPG (Fig. S5). Methane was below the detection limit at all times.

When the H_2_ production rate was low, YPG medium and BES were added to the bioreactor. After biogas generated in the bioreactor was removed using pure N_2_, the culture fluid was incubated at 55°C again without groundwater exchange and pH adjustment. In the second incubation, H_2_ was detected within 24 h and accumulated in the gas phase. The pressure increased to approximately 35 kPa in both bioreactors supplemented with 0.22% and 1.0% YPG. The H_2_ production operation was performed at least four times in the bioreactor using 0.22% YPG (Fig. 4) and at least three times in the bioreactor using 1.0% YPG (Fig. S5). The OD_600_ values for monitoring microbial biomass slightly increased. However, they were obviously low, compared with those in the bioreactors for CH_4_ production using same concentrations of YPG. The low microbial biomass in the bioreactor for H_2_ production is likely to be due to lack of H_2_/CO_2_-consuming methanogens, accumulation of fermentation products such as H_2_ and CO_2_, and a decrease in pH of the liquid phase.

The maximum H_2_ production rate was 6.08 mmol liter^−1^ day^−1^ in the bioreactor amended with 0.22% YPG, suggesting much higher than that in the bioreactor amended with 1.0% YPG (Table 1). There is a possibility that low pH in the bioreactor amended with 1.0% YPG affected the H_2_ production rate (Fig. S5).

### Microbial community in the bioreactor for H_2_ production

Bulk DNA was extracted from the microbial cells in the reactor for H_2_ production. Bacterial 16S rRNA genes were amplified by polymerase chain reaction (PCR), and a clone library was constructed. A total of 91 clones were randomly sequenced and divided into 10 OTUs (HYD-BR-B01 to HYD-BR-B10) (Table 2). The coverage of the clone library was 89%. Nearly 52% of clones in the clone library (HYD-BR-B01) were most closely related to the 16S rRNA gene from the anaerobic thermophilic bacterium *Thermotalea metallivorans* (99% similarity), which utilizes glucose for fermentation (Ogg and Patel, 2009). HYD-BR-B02 showed the highest identity to the 16S rRNA gene from the fermentative bacterium *T. syntrophicus* (83% similarity) (Sekiguchi *et al*., 2006), which accounted for 11% of all the clones in the library. HYD-BR-B03 showed the closest match to the 16S rRNA gene from the thermophilic bacterium *Schleiferia thermophila* (88% similarity), which was recovered from a hot spring (Albuquerque *et al*., 2011). The HYD-BR-B03 clones accounted for 9.9% of the clones in the library. HYD-BR-B04 and B05 were closely related to the 16S rRNA gene from the thermophilic sulfate-reducing bacterium *Desulfotomaculum putei* (99% and 89% similarity, respectively), which was collected from the deep terrestrial subsurface and utilizes H_2_, pyruvate, ethanol or lactate as electron donors (Liu *et al*., 1997). The OTUs accounted for 5.5% of all the clones in the library. The remaining low-abundance OTUs (HYD-BR-B06 to HYD-BR-B10) showed closest matches to the 16S rRNA genes from thermophilic anaerobic bacteria of the class *Clostridia* (Collins *et al*., 1994; Svetlitshnyi *et al*., 1996; Ogg and Patel, 2009).

**Figure 5:**
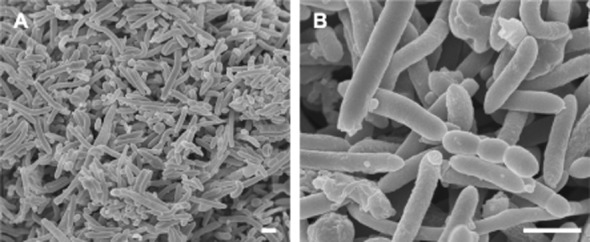
Scanning electron micrographs of microbial cells derived from the reactor for H_2_ production. Bar, 1 μm.

Scanning electron microscopy showed the dominance of cocci and relatively thick, sausage-shaped cells were observed frequently in the culture fluid for H_2_ production (Fig. 5A). We did not find the long cells and the flagellum-like filaments that were observed in the reactor for CH_4_ production (Fig. 5B). In the phylogenetic analysis, archaeal 16S rRNA gene was not amplified from the bulk DNA after repeated PCR. These results suggested that hydrogenotrophic methanogens derived from anaerobic groundwater were completely inhibited by BES supplementation.

Kimura and colleagues (2010) reported that hydrogenotrophic methanogens in the anaerobic groundwater used in this study were also inhibited by the incubation at high temperature of 70°C or 75°C, whereas the fermentative bacteria were able to maintain high activity and generate H_2_ under the culture condition. In the future, it should be possible to achieve economically advantageous H_2_ production by the high temperature incubation of anaerobic groundwater without the use of expensive BES.

## Experimental procedures

### Sampling site and environmental data analyses

An anaerobic groundwater sample was collected from a deep well (Ita-wari well; 34°52.3′N, 138°09.3′E) situated in Shimada, Shizuoka Prefecture, Japan (Fig. S1). This well is geologically located in the Setogawa group of the Paleogene accretionary prism, a group deposited 30–40 million years ago with abyssal sediment of the Nankai Trough (Tanabe and Kano, 1996). The deep well has been drilled down to 1489 m and constructed from tight steel casing pipes including strainers at 1188 to 1489 m. The groundwater is anaerobically drawn up to ground level by a water pump (Fig. S2).

Physical and chemical parameters of groundwater were measured at the outflow of the deep well. Temperature was measured with a platinum resistance thermometer (Custom, Tokyo, Japan). Oxidation-reduction potential and pH were measured with RM-20P and HM-20P portable meters (DKK-TOA, Tokyo, Japan) respectively. Electric conductivity was measured with a CM-21P portable meter (DKK-TOA). The anion and cation in the groundwater sample were analysed with an ICS-1500 ion chromatography system (Dionex, Sunnyvale, CA, USA). Dissolved organic carbon in the groundwater was measured with a TOC-V total organic carbon analyser (Shimadzu, Kyoto, Japan). Microbial cells in the groundwater were stained with SYBR Green I (1 : 100 dilution) (Life Technologies, Carlsbad, CA, USA), observed and counted under a model BX51 epifluorescence microscope (Olympus, Tokyo, Japan).

The concentration of dissolved natural gas was so high that gas was exsolving at the ground level (Fig. S2). The natural gas samples were collected in an inverted funnel underwater and then directed into autoclaved serum bottles. The serum bottles were tightly sealed with sterile butyl rubber stoppers and aluminum crimps underwater to prevent contamination by air. The concentrations of H_2_, N_2_, O_2_, CO_2_, CH_4_, ethane (C_2_H_6_) and and propane (C_3_H_8_) in the natural gas were determined on a GC-2014 gas chromatograph equipped with a thermal conductivity detector and flame ionization detector (Shimadzu).

### Bioreactor operation

A 12 litre capacity, stainless steel bioreactor was fabricated (Fig. 1A). The bioreactor was equipped with influent ports, sampling ports and a viewing window. The groundwater sample was anaerobically collected from the deep well and directly poured into the bioreactor through the influent port using a sterile silicone tube (Fig. S3). The anaerobic groundwater was overflowed for 15 min while being discharged from other influent port. Pure N_2_ was injected from the bottom sampling port that was mounted to the side of the bioreactor. At the same time, the groundwater sample was discharged from the middle sampling port mounted above the bottom sampling port. Finally, 5 litres of the liquid phase and 7 litres of the gas phase filled with pure N_2_ were anaerobically created in the bioreactor.

For methane production, organic substrates were autoclaved and added to the bioreactor. The organic substrates consisted of (per liter of water sample) 1.0 g of yeast extract (Bacto Yeast Extract: BD, Franklin Lakes, NJ, USA), 1.0 g of peptone (Bacto Peptone: BD) and 0.2 g of glucose (0.22% YPG) or 4.5 g of yeast extract, 4.5 g of peptone and 1.0 g of glucose (1.0% YPG). In the bioreactor for hydrogen production, 20 mM BES was also added. In order to completely remove dissolved natural gas from the groundwater sample, pure N_2_ was injected from the bottom sampling port while releasing gas from an upper sampling port mounted above the middle sampling port. The gas phase of the bioreactor was filled with pure N_2_ at 10 kPa in order to prevent contamination by air and microorganisms. These bioreactors for CH_4_ and H_2_ production were incubated at 55°C with a magnetic stirrer at 100 r.p.m. respectively (Fig. 1B). The incubation temperature was determined based on the highest activity of the microbial community that was found in enrichments incubated at 35, 45, 55, 65 and 75°C (data not shown).

The gas concentration, pressure, pH, and OD_600_ were measured every 24 h. The concentrations of H_2_, N_2_, CH_4_ and CO_2_ were determined using a GC-2014 gas chromatograph equipped with a thermal conductivity detector (Shimadzu). The pressure in the gas phase was measured with a KDM30 digital manometer equipped a needle (Krone, Tokyo, Japan). The pH of the liquid phase was measured with a B-212 Twin pH meter (Horiba, Kyoto, Japan). The abundance of microbial cells was monitored by measuring the OD_600_ using a GeneQuant 100 spectrophotometer (GE Healthcare, Bukinghamshire, UK). The acetate concentration was determined with an ICS-1500 ion chromatography system (Dionex).

When the biogas production rate became low, YPG (also BES for H_2_ production) was added to the bioreactor again. Then, the generated biogas was completely removed using pure N_2_ as described above. After the gas phase was filled with pure N_2_ at 10 kPa, the bioreactor was incubated at 55°C with a magnetic stirrer at 100 r.p.m.

### 16S rRNA gene analysis

Culture fluids were collected from the CH_4_-producing bioreactor amended with 1.0% YPG at day 20 and from the H_2_-producing bioreactor amended with 0.22% YPG and BES at day 9. Bulk DNAs were extracted as previously described (Ling and Liu, 2013). Briefly, cells were lysed using lysozyme and proteinase K solutions. Total nucleotide acid was extracted with successive phenol-chloroform-isoamyl alcohol and chloroform-isoamyl alcohol steps, and precipitated with ethanol. Ribonucleic acids were removed with RNase A solution. Archaeal and bacterial 16S rRNA genes were amplified by PCR from the bulk DNA using an *Archaea*-specific primer set, 109F and 915R (Kimura *et al*., 2013), and *Bacteria*-specific primer set, 8bF and 1512uR (Eder *et al*., 1999). Polymerase chain reaction products of the archaeal and bacterial 16S rRNA genes were cloned with a Zero Blunt TOPO PCR Cloning Kit for Sequencing with One Shot TOP10 *Escherichia coli* (Life Technologies). The sequences of inserted PCR products selected from recombinant colonies were determined with an Applied Biosystems 3730*xl* DNA Analyzer (Life Technologies).

The OTUs for each clone library were determined using GENETYX-Mac Ver. 17.0 (Genetyx, Tokyo, Japan). A 3% distance level between sequences was considered the cut-off for distinguishing distinct OTUs. The coverage of each clone library was calculated by the formula [1-(*n_1_*/*N*)], where *n_1_* is the number of OTUs represented by only one clone and *N* is the total number of clones examined (Good, 1953). The nearest relative of each OTU was determined by the blast program (Altschul *et al*., 1997).

### Scanning electron microscopy

Microbial cells for scanning electron microscopy were collected from the bioreactors at the same time as the samples for DNA extraction described above. The cells were fixed with 2% paraformaldehyde and 2% glutaraldehyde at 4°C overnight, and then with 1% tannic acid at 4°C for 2 h. The cells were then rinsed four times with 0.1 mM cacodylate buffer and fixed with 2% OsO_4_ at 4°C for 3 h. After the cells were dehydrated using a graded series of ethanol solutions, they were substituted into 100% tert-butyl alcohol and vacuumed dried. The resulting specimen was coated with a thin layer (50 nm) of osmium. The specimen was observed with a JSM-6340F scanning electron microscope (Jeol, Tokyo, Japan).

### Nucleotide sequence accession numbers

The 16S rRNA gene sequences obtained in this study were deposited in the DDBJ/EMBL/GenBank database under accession numbers AB910312 to AB910327.

## Conclusions

**Figure 6:**
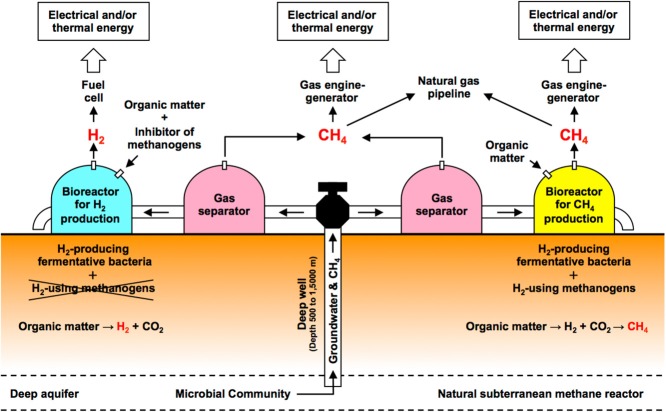
A novel biogas production system that combines the bioreactors for producing CH_4_ and H_2_ and a natural subterranean methane reactor in a deep aquifer associated with an accretionary prism.

This study demonstrated biogas production using the anaerobic groundwater collected from a deep aquifer of the accretionary prism in southwest Japan. Both CH_4_ and H_2_ were produced by a very simple incubation of anaerobic groundwater amended with organic substrates (also BES for H_2_ production) without pH and pressure adjustments. The phylogenetic analysis revealed the high diversity of microbial communities in the bioreactors for CH_4_ and H_2_ production, which is likely to suggest that the bioreactors have the ability to decompose various organic substrates and to produce biogas. The findings of the present study may be used for the development of ‘a novel bioenergy production system’ that combines the bioreactors for CH_4_ and H_2_ production and natural subterranean methane reactor in a deep aquifer associated with an accretionary prism (Fig. 6).

## Supporting Information

**Fig. S1.** Location of the sampling site in Shizuoka Prefecture, Japan. Areas of Cretaceous accretionary prism (black) and Paleogene-Neogene accretionary prism (gray) are shown in the geological map, which is taken from Kano and colleagues (1991). Broken lines indicate the convergent plate boundaries. ISTL, Itoigawa-Shizuoka Tectonic Line; MTL, Median Tectonic Line. **Fig. S2.** Photos of a deep well (A) and anaerobic groundwater and natural gas collected from the deep well (B). **Fig. S3.** Photos of a bioreactor, into which the groundwater sample is anaerobically poured using a sterile silicone tube (arrows). **Fig. S4.** Dynamics of the biogas and reactor in the bioreactor for CH_4_ production using anaerobic groundwater amended with 1.0% YPG: ▲, H_2_; ○, CH_4_; ×, CO_2_; ◇, pressure; □, pH; △, ODH_600_; ●, acetate. Gray lines indicate YPG supplement and biogas removal by bubbling using pure N_2_. **Fig. S5.** Dynamics of the biogas and reactor in the bioreactor for H_2_ production using anaerobic groundwater amended with 1.0% YPG-plus-20 mM BES: ▲, H_2_; ○, CH_4_; ×, CO_2_; ◇, pressure; □, pH; △, OD_600_; ●, acetate. Gray lines indicate YPG supplement and biogas removal by bubbling using pure N_2_. **Table S1.** Physical and chemical characteristics of anaerobic groundwater and natural gas obtained from deep well (Ita-wari).
