# Supplementary material for: Biogas production using anaerobic groundwater containing a subterranean microbial community associated with the accretionary prism
Source: Microb Biotechnol. 2014 Sep 29;8(5):837–45. doi: 10.1111/1751-7915.12179 (PMC4554471; doi:10.1111/1751-7915.12179)
Supplement: Supplementary file 1 [file mbt20008-0837-sd1.pdf]

Supporting Information

Biogas production using anaerobic groundwater containing a subterranean microbial community associated with the accretionary prism

Kyohei Baito[1], Satomi Imai[1], Makoto Matsushita[1], Miku Otani[1],

Yu Sato[1], Hiroyuki Kimura[1,2,3]

[1]Department of Geosciences, Graduate School of Science, Shizuoka University, Shizuoka, Japan.

[2]Center for Integrated Research and Education of Natural Hazards, Shizuoka University, Shizuoka, Japan.

[3]PRESTO, Japan Science and Technology Agency (JST), Kawaguchi, Saitama, Japan.

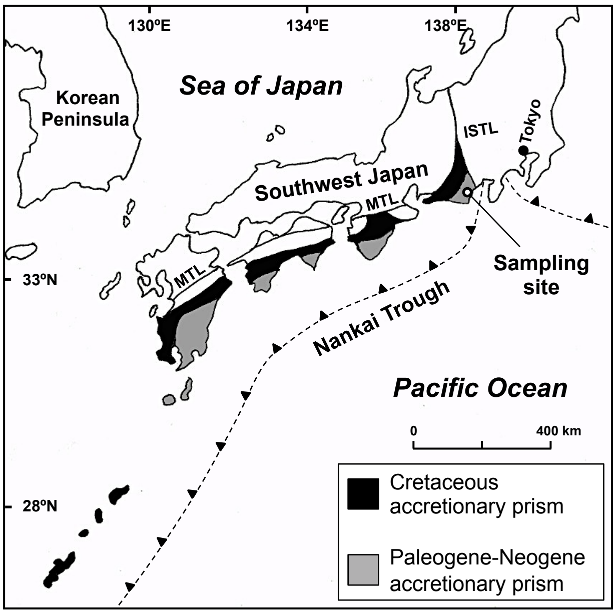

Fig. S1. Location of the sampling site in Shizuoka Prefecture, Japan. Areas of Cretaceous accretionary prism (black) and Paleogene-Neogene accretionary prism (gray) are shown in the geological map, which is taken from Kano *et al*. (1991). Broken lines indicate the convergent plate boundaries. ISTL, Itoigawa-Shizuoka Tectonic Line; MTL, Median Tectonic Line.

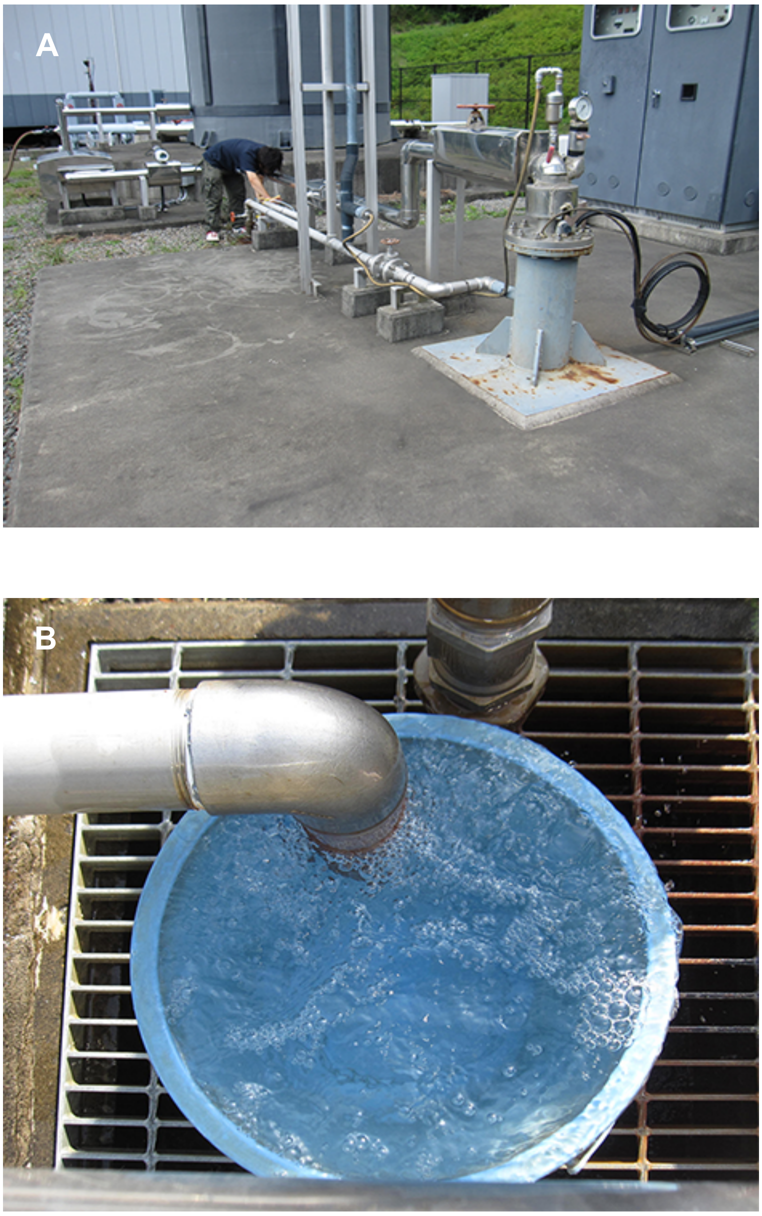

Fig. S2. Photos of a deep well (A) and anaerobic groundwater and natural gas collected from the deep well (B).

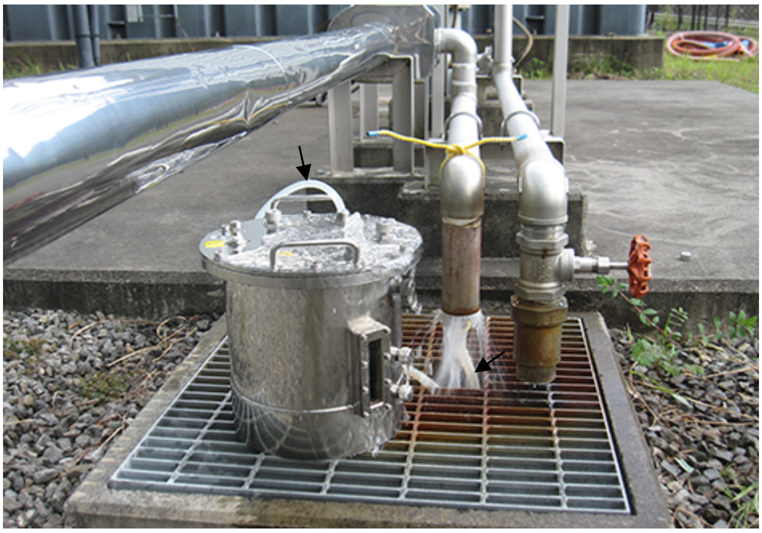

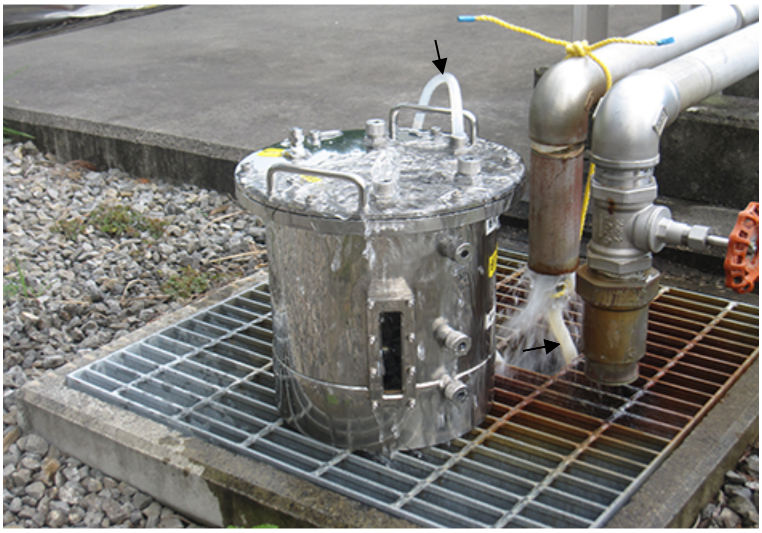

Fig. S3. Photos of a bioreactor, into which the groundwater sample is anaerobically poured using a sterile silicone tube (arrows).

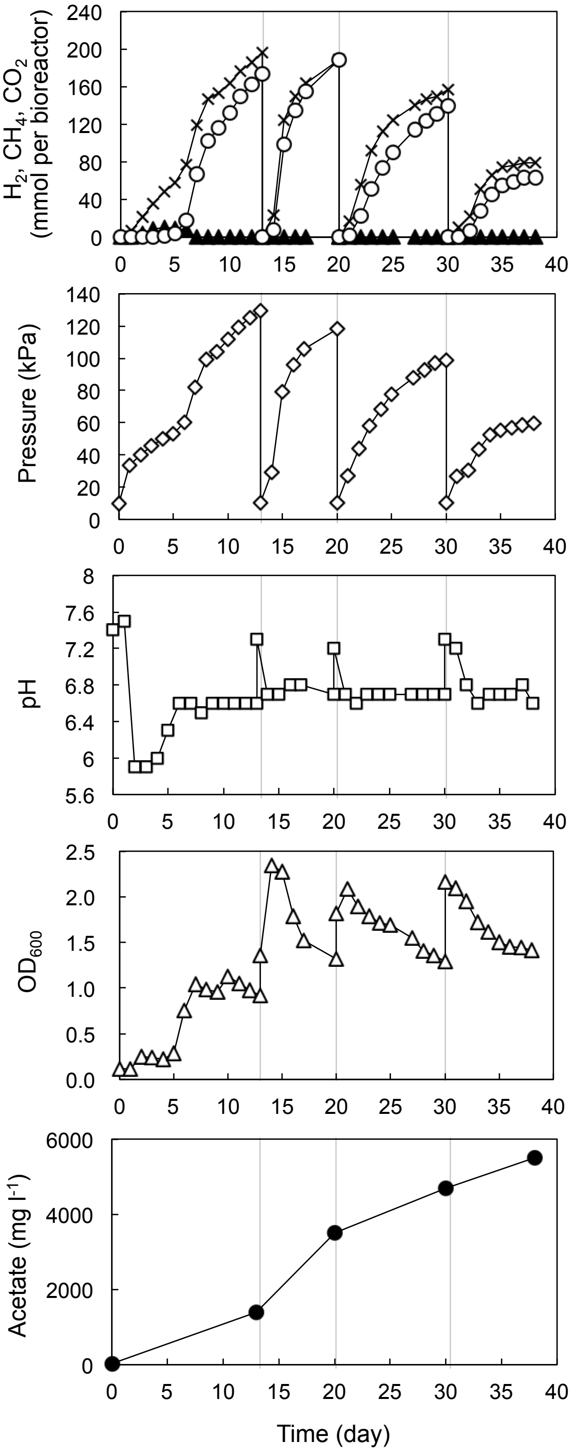

Fig. S4. Dynamics of the biogas and reactor in the bioreactor for CH_4 production using anaerobic groundwater amended with 1.0% YPG: ▲, H_2; ○, CH_4; ✕, CO_2; ◇, pressure; □, pH; △, OD_{600}; ●, acetate. Gray lines indicate YPG supplement and biogas removal by bubbling using pure N_2.

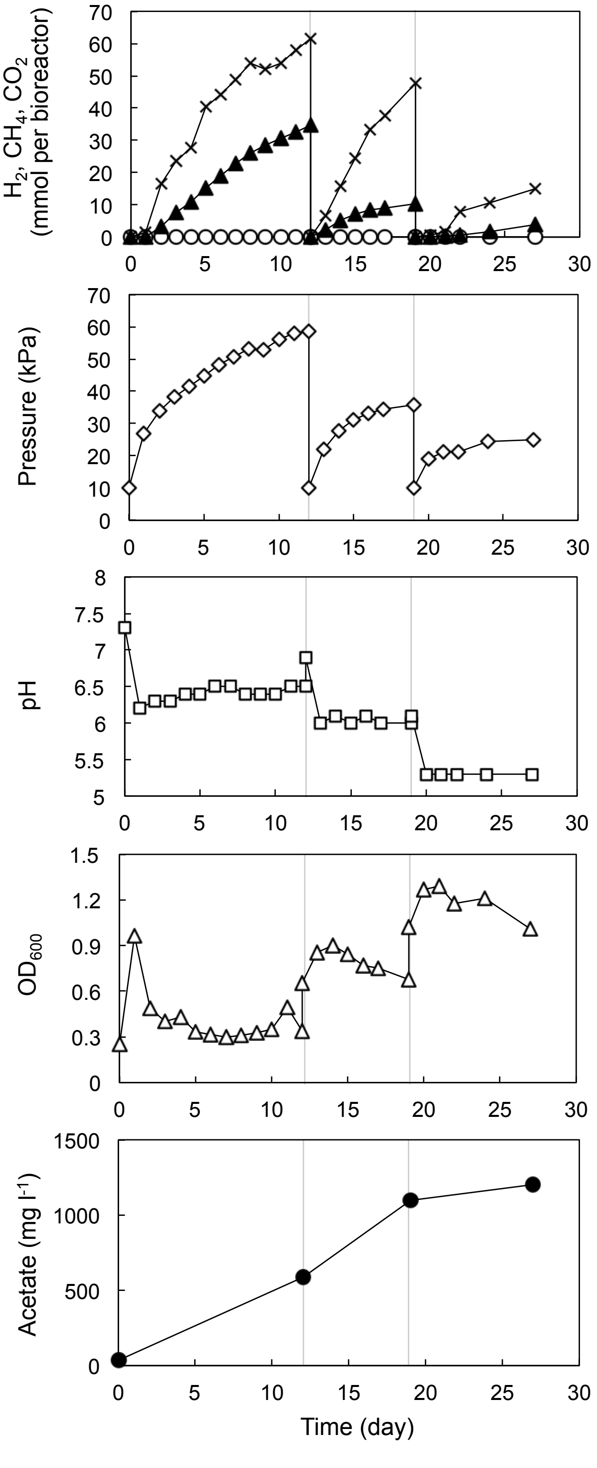

Fig. S5. Dynamics of the biogas and reactor in the bioreactor for H_2 production using anaerobic groundwater amended with 1.0% YPG-plus-20 mM BES: ▲, H_2; ○, CH_4; ×, CO_2; ◇, pressure; □, pH; △, OD_{600}; ●, acetate. Gray lines indicate YPG supplement and biogas removal by bubbling using pure N_2.

Table S1. Physical and chemical characteristics of anaerobic groundwater and natural gas obtained from deep well (Ita-wari).

Groundwater	Temperature	41.0	ºC
	pH	8.2	
	Oxidation-reduction potential (ORP)	-270	mV
	Electric conductivity (EC)	365	mS m^{-1}
	F^-	6.9	mg l^{-1}
	Cl^-	1,100	mg l^{-1}
	Br^-	0.5	mg l^{-1}
	I^-	3.0	mg l^{-1}
	S^{2-}	<0.01	mg l^{-1}
	NO_3^-	<0.1	mg l^{-1}
	SO_4^{2-}	0.8	mg l^{-1}
	PO_4^{3-}	<0.5	mg l^{-1}
	HCO_3^-	579	mg l^{-1}
	Acetate	<5.0	mg l^{-1}
	Formate	<1.0	mg l^{-1}
	Na^+	850	mg l^{-1}
	K^+	7.1	mg l^{-1}
	Mg^{2+}	<1.0	mg l^{-1}
	Ca^{2+}	7.5	mg l^{-1}
	NH_4^+	3.9	mg l^{-1}
	Dissolved organic carbon (DOC)	0.8	mg l^{-1}
Natural gas	H_2	<0.01	% (vol/vol)
	N_2	0.8	% (vol/vol)
	O_2	<0.01	% (vol/vol)
	CO_2	<0.01	% (vol/vol)
	CH_4	97.1	% (vol/vol)
	C_2H_6	2.1	% (vol/vol)
	C_3H_8	<0.01	% (vol/vol)
	C1/(C2+C3)	46	
